# Hemorrhoids

**Published:** 1898-02

**Authors:** 


### Hemorrhoids.

First, keep the liver in a condition to maintain a free supply of blood through it. For this purpose moderation in food and stimulants is imperative. Occasional small mercurial purgatives, followed by a mild saline, alum in small doses, and castor oil, do good. Exercise is useful in keeping the liver free, but this exercise must be of a certain kind. Both blood and bile have a tendency to stagnate in the liver; but this stagnation is lessened by the liver being rhythmically squeezed between the diaphragm and abdominal muscle. Brisk horseback exercise, either trotting or cantering, or touching the toes with the fingers, the knees being kept straight, is excellent exercise. A regular action of the bowels is of the utmost importance. The best time ordinarily for emptying the bowels is after breakfast, but if the piles have a tendency to come down, it is better to empty them every night before going to bed. The patient should take with him a bottle of hamamelis and some prepared sheep's wool. A small pledget of the wool should be inserted sufficiently to be grasped by the sphincters. This acts locally as a support to the piles, while hamamelis is healing and stops hemorrhages quickly.—*Public Health Journal.*

